# Preparation of Lignin-Based Phenolic Foam with Excellent Performance Based on Hydroxymethylation of Lignosulfonate and Paraformaldehyde

**DOI:** 10.3390/polym18131680

**Published:** 2026-07-07

**Authors:** Zhongbin Xu, Shushan Song, Xiang Zhen, Akram Ali Nasser Mansoor Al-Haimi, Zhongming Wang, Guocai Tian

**Affiliations:** 1 Faculty of Metallurgical and Energy Engineering, Kunming University of Science and Technology, Kunming 650093, China; 2 Guangzhou Institute of Energy Conversion, Chinese Academy of Sciences, Guangzhou 510640, China; 3 School of Chemistry and Chemical Engineering, Henan Normal University, Xinxiang 453007, China; 4 Dalian Institute of Chemical Physics, Chinese Academy of Sciences, Dalian 116023, China

**Keywords:** hydroxymethylation, lignosulfonate, phenol formaldehyde foam

## Abstract

In this paper, a novel biobased phenol formaldehyde resin foam was fabricated. Specifically, lignosulfonate, a byproduct of paper and pulping, is hydroxymethylated with paraformaldehyde and then condensed with phenol to form lignosulfonate-based phenol formaldehyde (LPF) resin, subsequently undergoing foam technology to prepare LPF foam. The structures and properties of the intermediate and target products were characterized by ^1^H nuclear magnetic resonance (^1^H NMR) spectroscopy, gel permeation chromatography (GPC), Fourier transform infrared spectroscopy (FT-IR), thermogravimetry derivative thermogravimetry (TGA-DTG), scanning electron microscopy (SEM), compression performance test, limiting oxygen index test and thermal conductivity measurement. It was found that the prepared foam exhibited excellent mechanical and thermal properties. At a lignin substitution degree of 10%, the optimal thermal stability (at 800 °C), compressive strength (0.14 MPa) and thermal conductivity (0.0294 W/m·K) were achieved. As the lignosulfonate content gradually increases, the limit oxygen index initially showed a significant increase and then decreased. It is worth noting that when the LS substitution degree is increased to 30%, the limiting oxygen index of foam is up to 32.6%. These results underscore the application potential of industrial lignin as a promising biobased substitute in the synthesizing PF foam with excellent thermal insulation and flame-retardant properties.

## 1. Introduction

Phenolic resins, which are typically prepared from phenols and formaldehyde, are good materials for a variety of applications because of their thermal, chemical, fire-proof, and dimensional stabilities. Generally, phenolic resins are commonly utilized in the wood industry as adhesives, paints, and varnishes, and also being employed in the aerospace, building, and construction sectors as composites and foams [1,2]. However, since the synthetic raw materials for phenolic resins (i.e., phenols and formaldehyde) are derived from petroleum resources [3], the discovery of new stocks has become more urgent owing to the depletion of such reserves. More specifically, it is necessary to develop new synthetic procedures or replace phenol with renewable phenolic substances to produce high-performance phenol formaldehyde (PF) resin foams [4,5,6].

In this context, many academics have focused on biomass as a renewable source of carbon reagents to replace petroleum resources. Indeed, owing to advancements in chemical technology, it is now possible to produce valuable chemicals and biofuels from biomass using a sustainable production approach [7]. For example, phenolic compounds, alcohols, and furans are just some of the chemicals produced by lignocellulose in biorefineries [8,9,10]. Lignin, a substantial by-product originating from the pulp and paper manufacturing sector, stands out as a readily available, cost-effective, sustainable, and innocuous biopolymer. Lignin is composed of three phenylpropyl alcohols, namely coniferyl alcohol (guaiacol), p-coumaryl alcohol (p-hydroxyphenylpropanol), and sinapyl alcohol (syringyl propanol) [11]. Although the phenolic nature of lignin has resulted in its widespread use for resin preparation, the irregular macromolecular structure and low reactivity have hindered its effective application [12].

The chemical modification is mostly applied to prepare phenolic resins from lignin. For example, hydroxymethylation [13], phenolation [14], and demethylation have been conducted to enhance the reactivity of lignin [15,16]. In our previous experimental studies, we attempted to synthesize lignin phenolic resins and foams by phenolic modification of lignosulfonate. However, we found that the introduction of lignin into the phenolic resin led to an increase in the free formaldehyde content. Although this can be addressed by the addition of urea at the end of the reaction to react with the free formaldehyde, the resulting urea–formaldehyde resin can affect the final performance of the phenolic resin and foam. We therefore sought to employ an alternative procedure to lower the generation of free aldehydes.

Unlike other biopolymers, the structural properties of lignin can change depending on its source plant species and processing methods. For example, when bagasse and sulfate lignin raw materials from the pulping process react with phenolic resin, the bonding strength of the phenolic resin increases and the gel time decreases [17]. In addition, through the addition of lignin extracted from wheat straw to phenolic resin using the organic solvent pretreatment method, the resulting products can meet various application requirements in terms of pH, viscosity, and solid content. However, when the lignin content of the phenolic resin reaches 50%, the curing temperature of the resin decreases [18,19]. Thus, in a previous study, we used lignosulfonate (LS) as the raw material to ensure the thermal stability of the PF resin foam and to control the resin viscosity and solid content [20]. Furthermore, intending to produce a more environmentally friendly process, we substituted the formaldehyde solution that is commonly used in the manufacture of hydroxymethylated ligninosulfonate (HLS) raw materials with paraformaldehyde powder. Upon increasing the scale of this procedure, we found that the formaldehyde content of the discharged wastewater was considerably reduced, which is consistent with the idea of “green chemistry”.

In this work, we proposed a new synthetic process of lignosulfonate-based phenol formaldehyde (LPF) resin foam with excellent properties. More specifically, lignosulfonates are hydroxymethylated with paraformaldehyde, which was then used as a feedstock to partially replace phenol and formaldehyde to prepare LPF resin. Subsequently, LPF resin foam was synthesized by a foaming technology. The characteristics of the modified lignosulfonate were detected by ^1^H NMR, GPC and FT-IR spectroscopy. In addition, the characterization of the structure and performance of foam involves FT-IR, TGA-DTG, SEM, compression performance testing, limiting oxygen index testing, thermal conductivity measurement and other technologies. The present work provides a new idea and method for the modification of lignosulfonate and the preparation of LPF resin foam.

## 2. Experimental Section

### 2.1. Materials

Lignosulfonate was procured from TCI-Tixi Ai (Shanghai) Chemical Industry Development Co., Ltd. (Shanghai, China), originating from the pulping black liquor of sodium sulfite, sourced from both coniferous and broadleaf tree species. Sodium hydroxide (NaOH), sulfuric acid (H_2_SO_4_), acetic anhydride, pyridine, and diethyl ether were purchased from Sinopharm Chemical Reagent Co., Ltd. (Shanghai, China). Additional reagents were sourced from Macklin Reagent Co., Ltd. (Shanghai, China). These reagents, of analytical purity, were employed without any subsequent purification.

### 2.2. Hydroxymethylation of LS

Paraformaldehyde (5 g), LS (1.5 g), NaOH (5 wt%), and water (10 mL) were mixed in a four-neck flask and stirred at 80 °C for 3.5 h. Subsequently, a small quantity of methanoic acid solution was introduced into the blend with the purpose of neutralizing the surplus sodium hydroxide. After that, the mixture was dissolved in 1, 4-dioxane (10 mL) and subsequently subjected to centrifugation at 5000 rpm for a duration of 5 min. The obtained supernatant was subsequently introduced into a volume of 500 mL of diethyl ether and then the precipitate was collected through centrifuging at 5000 rpm for 10 min. Finally, the resulting precipitate of HLS underwent a series of thorough washings employing diethyl ether to eliminate any residual paraformaldehyde, followed by an overnight freeze-drying process.

### 2.3. Preparation of LPF Resin

**Scheme 1:**
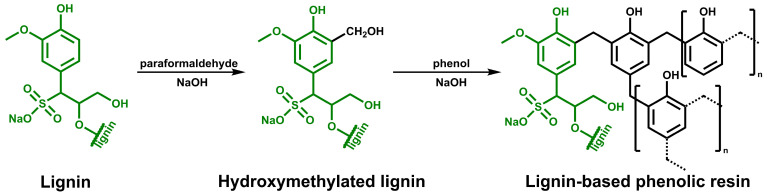
Synthetic route of lignin-based phenolic resin.

The first step of the preparation of LPF resins was the same as that described in Section 2.2, in which paraformaldehyde, water, LS, and NaOH were mixed (the detailed formulations were listed in Table 1) and reacted at 80 °C for 3.5 h (Scheme 1). After that, the pH of the system was adjusted to 9 by adding a methanoic acid solution to the mixture, and the resultant solution was cooled to 60 °C. After the addition of phenol, the polycondensation reaction was carried out at 60 °C for 1 h and 85 °C for 1 h. Finally, the black viscous oil was collected and marked as LPF resins.

### 2.4. Preparation of LPF Foam

Preceding the LPF foam preparation, an adjustment for the viscosity of the formulated LPF resin to achieve an extensible resin. In brief, LPF resin (10 g), n-pentane (1.5 g), and Tween-80 (0.6 g) were mixed and stirred for 30 s to form a homogeneous viscous oil and poured into an iron box mold. The reaction mixture was allowed to proceed as described above. Lastly, a quantity of 2.0 g of curing agent, comprising a 50% aqueous solution of p-toluenesulfonic acid and sulfuric acid (*v*/*v*, 1:1), were blended with the mixture (stirred for 30 s) and then poured into an iron box mold, which was placed in an oven at 85 °C for a duration of 15 to 20 min to be foamed.

### 2.5. Characterization

#### 2.5.1. Acetylation of the Lignosulfonate

LS (0.1 g) was dissolved in pyridine solution (1 mL), and then 1 mL of acetic anhydride was added and stirred at room temperature for 72 h (in darkness). The mixture was poured into 200 mL of deionized water [21] and the sediment was collected by centrifugation after being washed with deionized water (3–5×). The sample was then freeze-dried for 12 h and marked as acetylated LS.

#### 2.5.2. Gel Permeation Chromatography

The M_n_ (number-average molecular weight), M_w_ (weight-average molecular weight), and PDI (polydispersity index) of the lignin sample were obtained by Agilent PL-GPC220 (Agilent Technologies, Santa Clara, CA, USA; mobile phase: tetrahydrofuran; flow rate: 0.7 mL/min). Acetylated lignin, dissolved at a concentration of 5 mg/mL in N, N-dimethylformamide, was subjected to filtration through an organic membrane with a pore size of 0.45 μm. The establishment of a standard calibration curve was accomplished through the utilization of polystyrene reference standards.

#### 2.5.3. ^1^H Nuclear Magnetic Resonance Spectroscopy

The quantification of functional groups within the lignin samples was determined through the utilization of ^1^H NMR spectroscopy, employing an AVANCE III 400 MHz instrument by Bruker BioSpin (Rheinstetten, Germany). More specifically, the p-nitrobenzaldehyde (10 mg, internal standard) and acetylated lignin (50 mg) were added to DMSO-d_6_ (0.5 mL). By employing the integrated signal areas, the alcoholic hydroxyl (aliphatic OH), phenolic hydroxyl (PhOH), and methoxyl contents of LS could be calculated based on the following equations [22]:

(1) $$F_{methoxy}\left(mmol/g\;of\;LS\right)=\frac{\frac{I_{4.0-3.50\;ppm}}{3}\times \frac{2}{I_{8.10\;ppm}}\times \frac{m_{p-nitrobenzaldehyde}}{151}\times 1000}{m_{LS}-\frac{I_{2.50-1.70\;ppm}}{3}\times \frac{2}{I_{8.10\;ppm}}\times \frac{m_{p-nitrobenzaldehyde}}{151}\times 42}$$

(2) $$F_{PhOH}\left(mmol/g\;of\;LS\right)=\frac{\frac{I_{2.50-2.27\;ppm}}{3}\times \frac{2}{I_{8.10\;ppm}}\times \frac{m_{p-nitrobenzaldehyde}}{151}\times 1000}{m_{LS}-\frac{I_{2.50-1.70\;ppm}}{3}\times \frac{2}{I_{8.10\;ppm}}\times \frac{m_{p-nitrobenzaldehyde}}{151}\times 42}$$

(3) $$F_{aliphatic\;OH}\left(mmol/g\;of\;LS\right)=\frac{\frac{I_{2.27-1.70\;ppm}}{3}\times \frac{2}{I_{8.10\;ppm}}\times \frac{m_{p-nitrobenzaldehyde}}{151}\times 1000}{m_{LS}-\frac{I_{2.50-1.70\;ppm}}{3}\times \frac{2}{I_{8.10\;ppm}}\times \frac{m_{p-nitrobenzaldehyde}}{151}\times 42}$$

where F_PhOH_, F_Aliphatic OH_, and F_methoxy_ are the contents of PhOH, aliphatic OH, and methoxy in lignin (mmol/g), respectively. I_(2.27–1.70 ppm)_ and I_(2.50–2.27 ppm)_ denote the integrated regions of the acetyl group protons associated with the aliphatic OH and PhOH in lignin, respectively. I_(4.0–3.50 ppm)_ and I_(8.10 ppm)_ are the integrated areas of protons corresponding to methoxy (lignin) and aldehyde groups (p-nitrobenzaldehyde), respectively. M_(p-nitrobenzaldehyde)_ and MLS represent the masses of p-nitrobenzaldehyde (in grams) and LS (in grams), respectively. 151 and 42 refer to the relative molecular mass of p-nitrobenzaldehyde and acetyl group (with a subtraction of 1), respectively.

#### 2.5.4. Properties of LPF Resin and LPF Foam

The assessment of the viscosity, solids content, and free formaldehyde content of LPF resin adhered to the guidelines outlined in the Chinese National Standard (GB/T 14704-2017) [23]. Furthermore, the FT-IR spectrum of the LPF foam was obtained utilizing the instrument of a Nicolet IS50 (Thermo Fisher Scientific, Waltham, MA, USA), employing the KBr pellet technique. The wavenumber range and scan resolution were 400–4000 cm^−1^ and 4 cm^−1^, respectively. TGA was carried out on an SDT (TA Instruments, New Castle, DE, USA) to determine the thermal stability of LPF foam. The sample was heated from 25 °C to 800 °C at a rate of 10 °C/min under a nitrogen atmosphere (40 mL/min). After spraying gold on the surface of the sample, the surface area of LPF foam was observed by SEM using a Hitachi SU-70 (Hitachi High-Tech, Tokyo, Japan) at an accelerating voltage of 2.0 kV. A universal testing machine (Model 5565, Instron Corporation, Canton, OH, USA) was used to measure the compressive properties of LPF foams (i.e., at a deformation percentage of 20%) according to Chinese National Standard (GB/T 1041-2008) [24], where the specimen (50 mm × 50 mm × 25 mm) was tested between stainless-steel plates, and the strain rate was set to a value of 2.5 mm/min. The thermal conductivity of LPF foam (rectangular specimen: 20 mm × 20 mm × 3 mm) was evaluated on a hot disk TPS 2500S (Hot Disk AB, Göteborg, Sweden) according to ISO-22007-2:2022 [25], which was performed employing the transient planar heat source technique at room temperature. The determination of the limiting oxygen index for the LPF foam (with a dimension of 100 mm × 10 mm × 5 mm) was measured on an FTT0077 (Fire Testing Technology Limited, East Grinstead, UK) according to the Chinese National Standard (GB/T 2406.5-2025) [26]. At least three specimens were tested for each formulation, and the average values were adopted for analysis.

## 3. Results and Discussion

### 3.1. Characterization of Lignin

**Figure 1:**
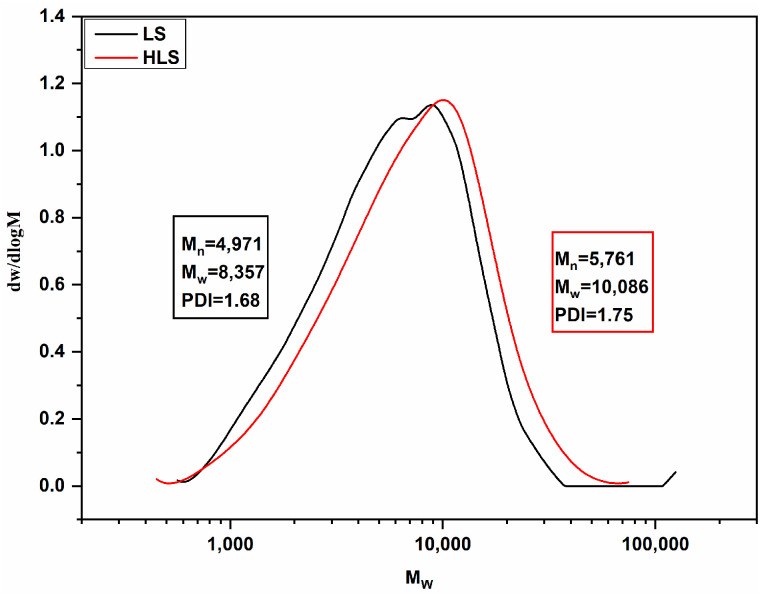
Molecular weight distribution curves for the LS and HLS specimens.

Figure 1 depicts the changes in molecular weight distribution of LS specimens after modification. It can be seen that the M_w_, M_n_, and PDI were increased from 8375 g/mol, 4971 g/mol, and 1.68 to 10,086 g/mol, 5761 g/mol, and 1.75, respectively. It indicated that formaldehyde had been successfully introduced into the lignin molecular system. According to the literature, under alkaline conditions, lignin and formaldehyde can undergo hydroxymethylation reactions on the aromatic ring of lignin and the side chain of the aromatic ring, thereby improving the reactivity of lignin [27]. Therefore, although some lignin undergoes condensation reaction, hydroxymethylation increases the content of active crosslinking sites in lignin.

**Figure 2:**
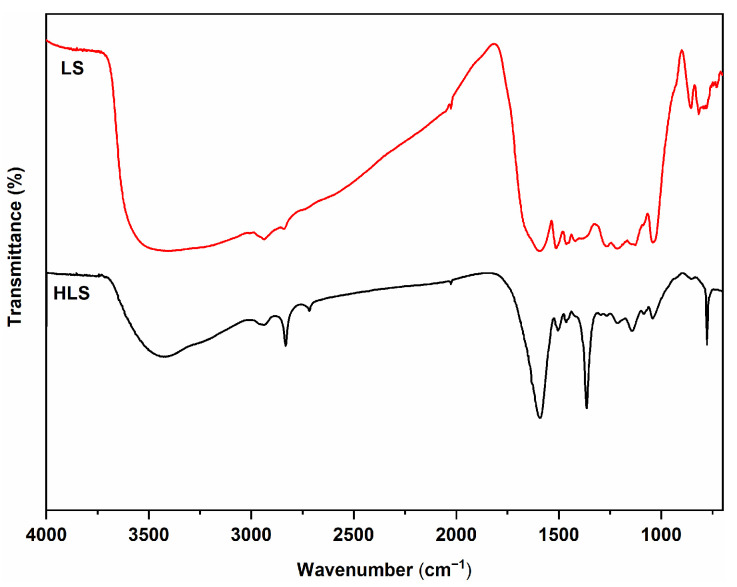
FT-IR spectra of the LS and HLS specimens.

To further confirm the hydroxymethylation of LS, the LS and HLS specimens were subjected to FT-IR measurements, and the characteristic peak positions and corresponding functional groups are listed in Figure 2 and Table 2 [28]. As shown in Figure 2, several main absorption peaks observed for HLS and LS were similar, indicating that lignin retains its original structural groups to a large extent after hydroxymethylation. In addition, it can be seen that during the hydroxymethylation reaction, the characteristic peaks at 3400, 2900, and 1030 cm^−1^ were significantly enhanced, which confirms that the hydroxymethyl content gradually increased.

**Figure 3:**
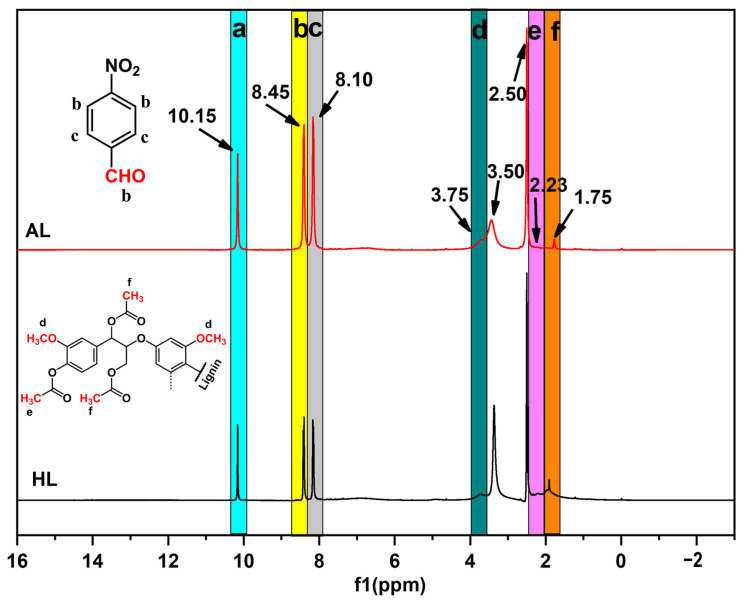
Typical ^1^H NMR spectra recorded for LS and HLS.

Quantitative measurements of alterations in the functional group contents, including aliphatic OH, PhOH, and methoxy groups, within the lignin sample were conducted through the utilization of ^1^H NMR spectroscopy, as depicted in Figure 3. The proton NMR spectrum displayed a signal at 10.15 ppm (peak a), indicative of the aldehyde proton within 4-nitrobenzaldehyde. Furthermore, the signal at 8.45 ppm (peak b) was attributed to the nitro-ortho-phenyl ring, and the signal at 8.10 ppm (peak c) represented the aldehyde-ortho-phenyl ring. Moreover, within the spectrum, the signals spanning the range of 6.5–7.5 ppm (peak d) were ascribed to the benzene ring protons of LS, while the robust proton signals within the range of 3.50–4.00 ppm (peak e) could be attributed to the methoxy groups of LS. Additionally, the peaks at 2.27–2.50 ppm (peak f) and 1.70–2.27 ppm (peak g) corresponded to the PhOH groups and the aliphatic OH groups of LS, respectively.

In order to assess the influence of phenolic modification on the lignin structure, variations in the PhOH, aliphatic OH, and methoxy group contents between LS and HLS were quantitatively determined. As presented in Table 3, the modification had a significant effect on the contents of these functional groups. In detail, there was a substantial increase in the concentrations of aliphatic OH and PhOH groups, which elevated from 2.328 and 0.954 mmol/g to 3.723 and 2.295 mmol/g, respectively, whereas the content of the methoxy group was significantly decreased from 2.901 to 1.830 mmol/g. Overall, these results demonstrated that the content of reactive groups (aliphatic OH and PhOH) had a significant increase in the HLS structure (c.f., the LS structure), which is more conducive to the subsequent use of HLS to synthesize LPF resin and foam.

### 3.2. Characterization of LPF Resins

The viscosity of the LPF resin serves as an indicator of the extent of condensation. It is important to emphasize that a low resin viscosity may result in premature release of the blowing agent, whereas a high viscosity can impede the effective blending and expansion of the resin. Both scenarios of low and high viscosity can consequently result in foaming failure [29]. Hence, adequate viscosity is also crucial for successful foaming. As outlined in Table 4, upon increasing the LS content of the phenolic resin, the viscosity of the LPF resin gradually increased, which was attributed to the substantially greater molecular weight of the lignin component in the resin synthesis process.

The concentration of free formaldehyde within the LPF resin is documented in Table 4. It is evident from the results that an augmentation in LS content, ranging from 10% to 40%, yielded a concomitant decrease in the free formaldehyde content, reducing it from 0.140% to 0.067%. This can be attributed to the fact that LS combines with paraformaldehyde during the hydroxymethylation reaction to reduce the free formaldehyde content of the final resin. However, when the LS content reached 50%, the free formaldehyde content was slightly increased. This may be due to the saturation of available active sites on LS for unable to react with formaldehyde. Interestingly, although the free formaldehyde content of the resin increased when the LS content reached 50%, it was still significantly lower than the corresponding value for the PF resin, which is in line with the concept of green chemistry.

### 3.3. Characterization of LPF Foams

#### 3.3.1. Structure Analysis of LPF Foams

**Figure 4:**
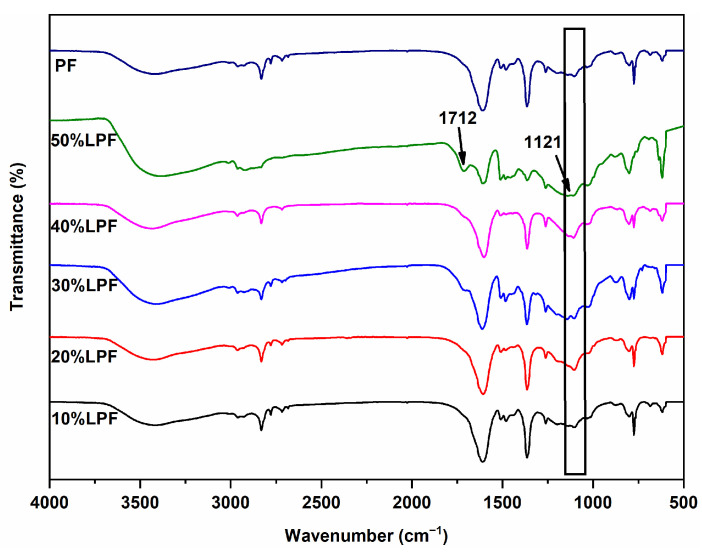
FT-IR spectra of LPF foams with different LS contents.

As depicted in Figure 4, the infrared spectra of the LPF foams exhibit resemblances to those of PF foams, although minor deviations in signal intensities were discernible. These results indicate that lignin-based phenolic foams possess similar molecular structures to those of phenolic foams. More specifically, the prepared foams exhibited broad and strong peaks corresponding to the hydroxyl vibrations (3300–3400 cm^−1^) and the C–H in-plane vibrations of the methyl and methine groups (2850–2930 and 755–852 cm^−1^, respectively), wherein the latter peaks specifically correspond to the bending vibrations at the –CH planes of the disubstituted benzene and trisubstituted benzene species, respectively. These observations indicate that phenolic polymerization of ortho- and para-substituted benzene rings occurred during the synthesis of LPF. It is worth noting that due to the hydroxymethylation of LS, the corresponding hydroxymethyl signal at ~1121 cm^−1^ was significantly enhanced in the spectra of the LPF foams compared to that of the PF foam. Moreover, in the case of the LPF foam containing 50% LS, strong vibrational peaks were observed at ~1710 cm^−1^, corresponding to unconjugated ketones and other carbonyl groups. This signal is generally not present in the spectrum of phenolic resin, indicating that when the degree of lignin replacement reached 50%, blending of the lignin and the phenolic resin occurred.

#### 3.3.2. Thermal Stability of LPF Foams

**Figure 5:**
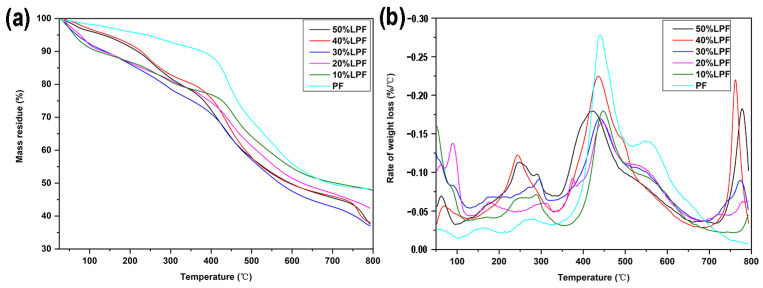
(**a**) TGA and (**b**) DTG curves for LPF foams.

In Figure 5, the TG-DTG curves of the LPF foams, developed through varying degrees of lignin substitution, are presented. Notably, an increase in lignin content correlated with a discernible reduction in the thermal stability of the foams. In addition, similar thermal decomposition processes were observed for LPF and PF foams, with three distinct decomposition stages occurring at temperature ranges <350 °C (stage I), 350–650 °C (stage II) and 650 to 800 °C (stage III), respectively. In stage I, the weight loss is attributed to the evaporation of free formaldehyde, water, phenol, and small molecules of phenolic-modified lignin [30]. During stage II, the ether linkage is cleaved, yielding more stable methylene bridges and releasing water. Simultaneously, the phenolic constituents undergo further degradation, forming carbonaceous structures. In stage III, the foam structure and methylene bridge are destroyed.

As evident from Figure 5, the augmented lignin content correlates with the onset of lignin skeleton degradation occurring in the temperature range of 250 to 350 °C. Concurrently, the decomposition of numerous side chains becomes noticeable. As a result, a greater degree of weight loss was observed in this stage for the LPF foam compared to subsequent stages. Nevertheless, the structural integrity of the foam remained unaltered throughout this thermal decomposition process, underscoring the notably high thermal stability of the foam material. At 800 °C, the weight loss rate was essentially negligible, and the char yield was greater than 35%, which further indicates that the introduction of lignin improves the thermal stability of the foam.

#### 3.3.3. Morphological Analysis of LPF Foams

**Figure 6:**
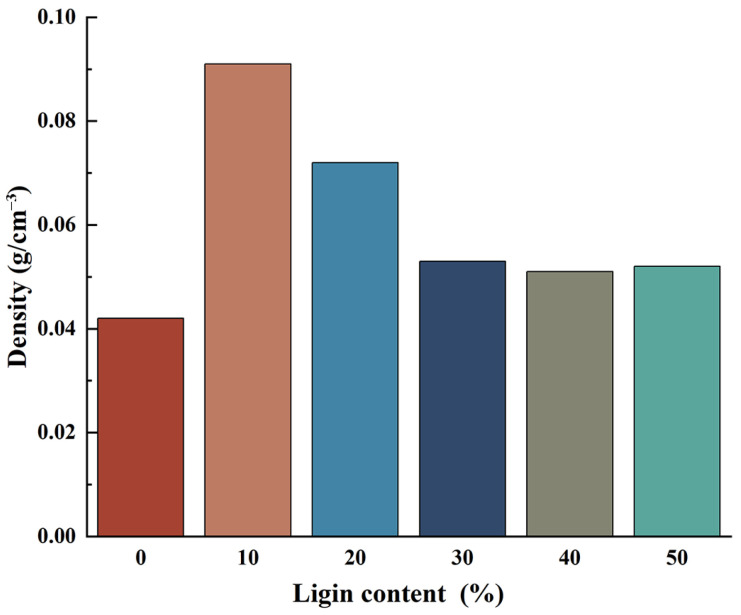
Apparent density curve of LPF foam under different LS contents.

**Figure 7:**
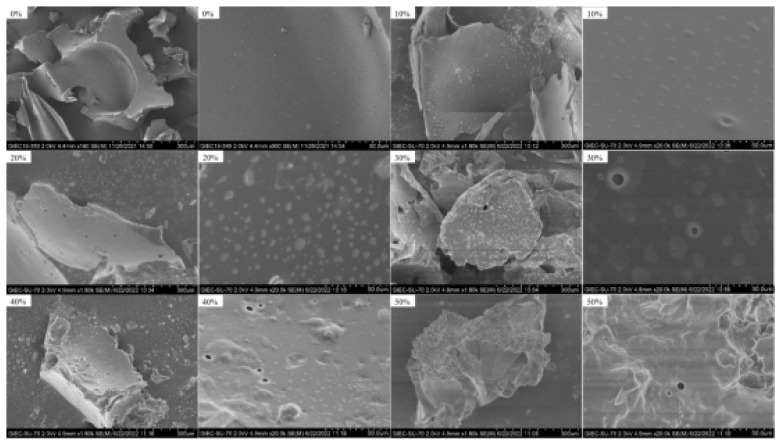
SEM images of LPF foam powder with different LS contents.

The foam density is a key parameter responsible for determining the performance and application of phenolic foams. Figure 6 lists the densities of LPF foams prepared with different LS contents. It shows that the foam density decreased with an increase in LS content. It should be noted that a foam density range of 0.04–0.09 g/cm^3^ is generally suitable for preparing thermal insulation and flame-retardant materials. Notably, the introduction of lignin into the phenolic foam matrix resulted in a foam with a higher density compared to the original PF foam. This outcome can be ascribed to the elevated molecular weight of lignin, which contributes to an overall increase in foam density. However, as the LS content was progressively raised, the reactivity of the LPF resin diminished, leading to a more rigid lignin structure that amplified foam porosity. Consequently, this structural alteration ultimately yielded a larger foam volume and, consequently, a reduced foam density. Interestingly, when the LS content is 50%, the density of foam slightly increases, which may be attributed to the fact that when the internal crosslinking of the resin is completed under the same foam volume, the quality of some unreacted blocky LS is higher, leading to a slight increase in density. This problem can also be observed in the SEM images presented in Figure 7.

More specifically, upon examination of the high magnification SEM images, upon increasing the LS content, the initially smooth foam surface became rough, and particle formation can be observed at LS contents >40%. These particles are likely composed of lignin. Hence, with lignin concentrations of 10%, 20%, and 30%, the anticipated outcome was a uniform dispersion of lignin within the LPF foam through crosslinking with PF. Consequently, the foam surface exhibited a smooth texture with minimal voids. However, as the lignin content was elevated to 40% and 50%, it became evident that certain agglomerated lignin aggregates adhered to the foam surface. This phenomenon was attributed to the insufficient availability of crosslinking sites within the resin matrix for accommodating the increased lignin content. Thus, when present in the appropriate proportion, the addition of LS to PF foam can potentially toughen PF resin foam through crosslinking, as examined in the following section.

#### 3.3.4. Compressive Performance of LPF Foams

**Figure 8:**
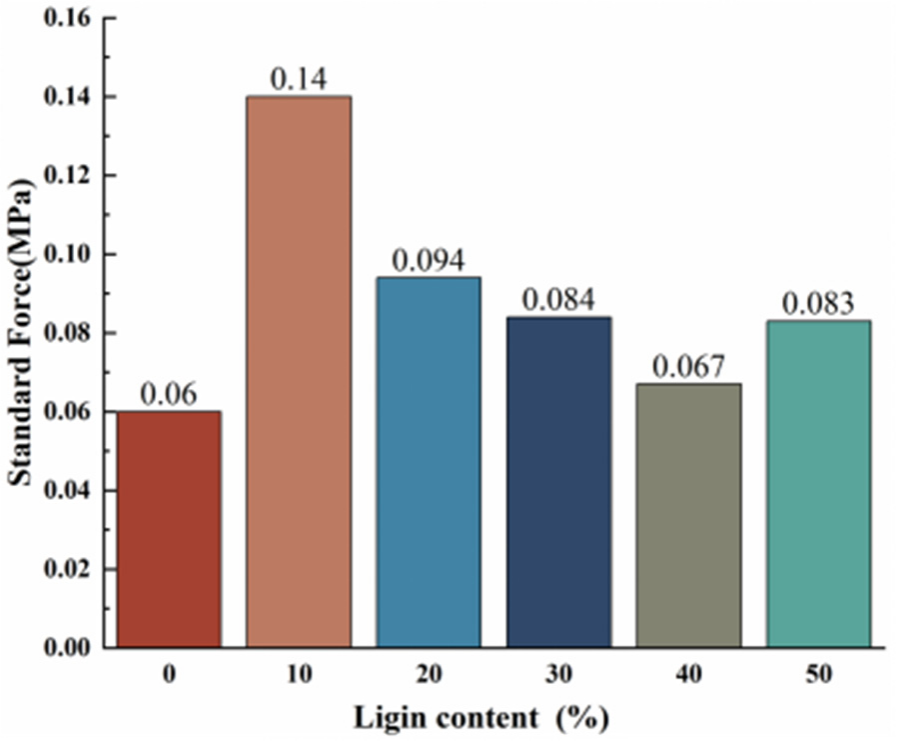
Effect of LS content on the compressive strengths of LPF foams.

To evaluate the performances of the prepared LPF foams, the effect of LS content on the compressive strength was initially investigated. As shown in Figure 8, the compressive performance of the foam was improved with the addition of LS. The large molecular mass and numerous linear alkyl side chains of lignin can contribute to a superior compressive strength for foam formation [31]. In addition, paraformaldehyde rather than formaldehyde was employed during the hydroxymethylation process. The linear structure of polyoxymethylene provides intramolecular flexibility to improve the compression resistance of the foam. It is worth noting that the compressive strength of LPF foam with a LS content of 10% reached 0.14 MPa, which is a significantly larger value than that achieved for the PF foam. As illustrated in Figure 6, the 10% LPF sample also exhibits the maximum foam density among all formulations. FTIR and SEM characterizations verify that covalent crosslinking were formed between lignosulfonate and PF matrix at lignin substitution ratios below 20%. These results demonstrate that the enhanced compressive strength originates from two synergistic factors: the crosslinking network serves as the primary reinforcement contributor, while the variation in foam density offers auxiliary mechanical improvement.

**Figure 9:**
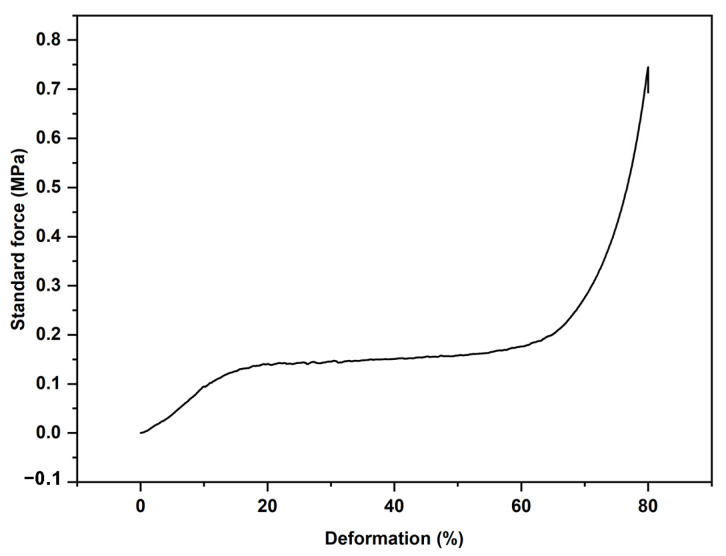
Relationship between the compressive deformation and the compressive strength of 10LPF foam.

To investigate the compressive ability of 10%LPF foam more thoroughly, the relationship between the compressive deformation and the compressive strength of this specimen was examined. As can be seen from Figure 9, the compressive capacity of the 10%LPF foam is excellent, with a compressive strength of 0.745 MPa being reached at a compressive deformation of 80%. These results, therefore, serve as a solid cornerstone for the future industrial application of LPF foams.

#### 3.3.5. Thermal Conductivity of LPF Foams

**Figure 10:**
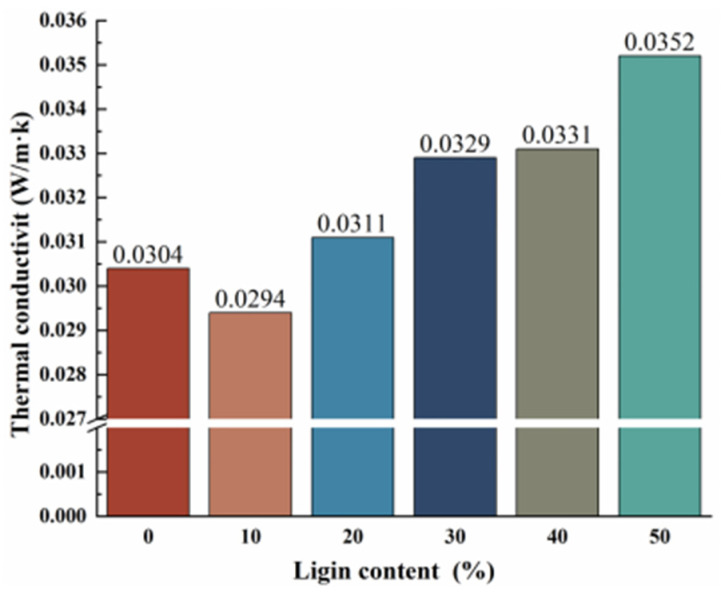
Thermal conductivities of LPF foams containing different LS contents.

Generally, materials with a low thermal conductivity (i.e., ≤0.12 W/(m·K) at an average temperature < 350 °C) are known as thermal insulators, while those with a thermal conductivity of 0.05 W/(m·K) are known as high-efficiency thermal insulation materials. In this context, we note that the thermal conductivities of currently available PF foams range from 0.03 to 0.05 W/(m·K). In our case (Figure 10), it was found that only a small increase in the thermal conductivity of the foam took place upon increasing the lignin content. It was therefore considered that the addition of lignin does not have any significant effect on the cell structure of the phenolic foam, and thus, the thermal conductivity remains relatively constant. This observation suggests that the closed-cell ratio within the foam structure is elevated. To elaborate, during the heat transfer process of a foam material, the enclosed gas molecules within these closed cells exhibit poor thermal conductivity properties. Consequently, heat transfer transpires at a notably sluggish pace, thereby leading to low thermal conductivity of the material. Compared with the literature (Table 5), it appears that the prepared LPF resin foams have the potential to be used as good thermal insulation materials.

#### 3.3.6. Limiting Oxygen Index of LPF Foams

**Figure 11:**
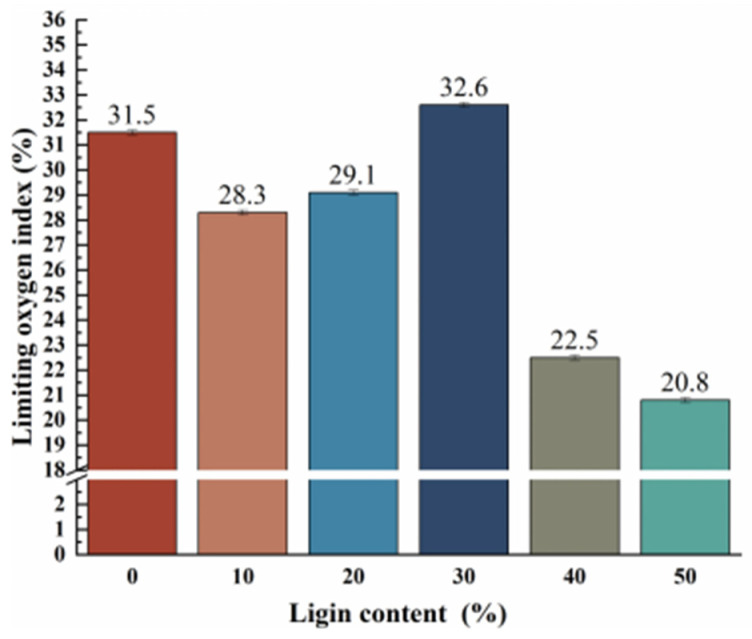
Influence of the lignin content on the limiting oxygen index of LPF foam.

It is generally considered that materials with oxygen indices <21 are flammable, those between 21 and 27 are flame-retardant, and those >28 are flame-retardant. As shown in Figure 11, upon increasing the lignin content of the prepared LPF foams, the limiting oxygen index initially increased prior to decreasing. In addition, with the exception of the 30%LPF specimen, the oxygen indices of these LPF foams were lower than those of ordinary phenolic foams. It was potentially attributed to the incomplete depolymerization of paraformaldehyde and subsequent polycondensation during the hydroxymethylation of lignin. As a result, a flammable flexible chain is introduced into the foam structure, the proportion of aromatic rings is reduced, and the consumption of oxygen increases during the combustion process, thereby reducing the flame-retardant performance. Moreover, with an increase in the lignin content, a large number of alkyl branches are introduced into the foam structure, which also leads to a less rigid structure during combustion. It should also be noted that the raw lignin material contains sugar molecules, among other components, and as a result, the presence of these components may potentially diminish the flame-retardant characteristics of the foam material.

## 4. Conclusions

In summary, we have presented an efficient methodology for the fabrication of lignin phenol-formaldehyde (LPF) foams via the foaming process of an LPF resin. More specifically, lignosulfonates (LS) were hydroxymethylated with paraformaldehyde (giving HLS) and then condensed with phenol to obtain the desired resin. More specifically, the prepared HLS was used to replace the phenol raw material (≤50% HLS) in the condensation reaction with formaldehyde. Subsequently, the LPF resin underwent a foaming procedure, resulting in the formation of the corresponding LPF foam. Thus, LS specimens were analyzed by Fourier transform infrared spectroscopy, gel permeation chromatography, and ^1^H nuclear magnetic resonance spectroscopy before and after hydroxymethylation modification, and the results of these investigations confirmed the feasibility of this procedure. It was found that HLS is more reactive than LS under alkaline conditions, and its incorporation can significantly reduce the content of the carcinogenic free formaldehyde in the final resin. Furthermore, the prepared foam exhibited excellent mechanical and thermal properties, with optimal thermal stability (at 800 °C) and compressive strength (0.14 MPa) being obtained with a lignin substitution degree of 10%. With the progressive augmentation of LS content, ranging from 10% to 50%, there was a marginal increase in thermal conductivity. Notably, the limiting oxygen index exhibited an initial substantial increase, followed by a subsequent decline. Moreover, with a LS content of 10%, the thermal conductivity of the foam reached a minimum of 0.0294 W/m·K, and when the degree of LS substitution was increased to 30%, the limiting oxygen index of the foam reached a maximum of 32.6%, thereby indicating the potential of LPF resins for application in thermally insulating and flame-retardant materials.

## Figures and Tables

**Table 1:** Formulation of LPF resins with different substitution degrees.

Substitution Degree (%)	Lignin (g)	Phenol (g)	Paraformaldehyde (g)	NaOH (g)	Water (mL)	Formic Acid (mL)
0	0	33.6	15	0.3	0	0
10	2.5	22.9	10.2	0.6	15	0.3
20	5.1	20.3	9.1	0.7	15	0.4
30	7.6	17.8	7.9	0.8	15	0.5
40	10.2	15.3	6.8	0.9	15	0.7
50	13.7	13.7	6.1	1.0	15	0.7

**Table 2:** FTIR absorption peak location and assignments.

Wavenumber (cm^−1^)	Peak Location and Assignment
3300–3400	–OH stretching
2850–2930	–C–H– stretching
1700	C=O stretching of carbonyl and aliphatic groups
1612	Aromatic skeletal stretching and C=O stretching
1514	Aromatic skeletal stretching
1464	C–H deformation
1428	Aromatic skeletal stretching combined with C–H in-plane deformation and stretching
1210	Syringyl ring and C–H stretching
1032	C–H stretching
757	Aromatic skeletal stretching

**Table 3:** The content of different functional groups in LS and HLS determined by ^1^H NMR spectroscopy.

Lignin Structure	PhOH (mmol/g)	Aliphatic OH (mmol/g)	Methoxy (mmol/g)
LS	2.328	0.954	2.901
HLS	3.723	2.295	1.830

**Table 4:** Properties of LPF resins with different LS contents.

Resin	Viscosity (mPa s^−1^)	Solid Content (%)	Free Formaldehyde (%)
PF	935	75.6	0.201
10%LPF	1100	75.9	0.140
20%LPF	1350	75.7	0.094
30%LPF	1650	73.3	0.084
40%LPF	2870	71.9	0.067
50%LPF	3750	70.6	0.083

**Table 5:** Comparison of different properties with reported LPF foams.

Lignin	Modification Method	Thermal Conductivity (W·m^−1^ k^−1^)	Compressive Strength (MPa)	Refs.
Soda lignin	phenolation demethylation	0.028	0.25	[32]
Sodium Ligninsulfonate	phenolation	0.047	0.12	[33]
Enzymatic hydrolysis lignin	phenolation	0.035	0.35	[34]
Acid hydrolysis lignin	phenolation	0.025	0.33	[35]
Enzymatic hydrolysis lignin	nitrogenization phosphorization	0.032	0.21	[36]
Sodium Ligninsulfonate	phenolation	0.034	0.24	[37]
Soda lignin	demethylation	0.032	0.23	[38]
Alkaline lignin	phenolation	0.042	0.11	[39]
Sodium Ligninsulfonate	hydroxymethylation	0.029	0.14	This work

## Data Availability

The original contributions presented in this study are included in the article. Further inquiries can be directed to the corresponding authors.

## References

[1] Sarika P.R., Nancarrow P., Khansaheb A., Ibrahim T. (2020). Bio-based alternatives to phenol and formaldehyde for the production of resins. Polymers.

[2] Lazko J., Mariage J., Joyet C., Layachi A., Satha H., Dubois P., Laoutid F. (2026). Toward safer and greener insulation: Formaldehyde-free, flame-retardant, and bio-based phenolic foams from tannin and modified-lignin combination. Materials.

[3] Knop A., Pilato L.A. (1985). Phenolic Resins: Chemistry, Applications and Performance.

[4] Li B., Wang Y., Mahmood N., Yuan Z., Schmidt J., Xu C.B. (2017). Preparation of bio-based phenol formaldehyde foams using depolymerized hydrolysis lignin. Ind. Crop. Prod..

[5] Liu W.-J., Jiang H., Yu H.-Q. (2015). Thermochemical conversion of lignin to functional materials: A review and future directions. Green Chem..

[6] Lora J.H., Glasser W.G. (2002). Recent industrial applications of lignin: A sustainable alternative to nonrenewable materials. J. Polym. Environ..

[7] Asim M., Saba N., Jawaid M., Nasir M., Pervaiz M., Alothman O.Y. (2018). A review on phenolic resin and its composites. Curr. Anal. Chem..

[8] Aziz N.A., Latip A.F.A., Peng L.C., Latif N.H.A., Brosse N., Hashim R., Hussin M.H. (2019). Reinforced lignin-phenol-glyoxal (LPG) wood adhesives from coconut husk. Int. J. Biol. Macromol..

[9] Ballerini A., Despres A., Pizzi A. (2005). Non-toxic, zero emission tannin-glyoxal adhesives for wood panels. Eur. J. Wood Wood Prod..

[10] Binetti R., Costamagna F.M., Marcello I. (2006). Development of carcinogenicity classifications and evaluations: The case of formaldehyde. Ann. dell’Istituto Super. Sanità.

[11] Dutta S., De S., Saha B. (2013). Advances in biomass transformation to 5-hydroxymethylfurfural and mechanistic aspects. Biomass Bioenergy.

[12] Li B., Yuan Z., Schmidt J., Xu C.B. (2019). New foaming formulations for production of bio-phenol formaldehyde foams using raw kraft lignin. Eur. Polym. J..

[13] Ai X.B., Feng S.H., Shui T., Kakkar H., Xu C.B. (2021). Effects of alcell lignin methylolation and lignin adding stage on lignin-based phenolic adhesives. Molecules.

[14] Heo J.B., Lee Y.-S., Chung C.-H. (2019). Raw plant-based biorefinery: A new paradigm shift towards biotechnological approach to sustainable manufacturing of HMF. Biotechnol. Adv..

[15] Hirayama T., Yamada N., Nohara M., Fukui S. (1984). The high performance liquid chromatographic determination of total malondialdehyde in vegetable oil with dansyl hydrazine. J. Sci. Food. Agr..

[16] Hirano K., Asami M. (2013). Phenolic resins—100 years of progress and their future. React. Funct. Polym..

[17] Zhou J., Hu L., Liang B., Bo C., Jia P., Zhou Y. (2015). Preparation and characterization of novolac phenol-formaldehyde resins with enzymatic hydrolysis lignin. J. Taiwan Inst. Chem. Eng..

[18] Kuster B.F.M. (2010). 5-Hydroxymethylfurfural (HMF): A review focussing on its manufacture. Starch-Starke.

[19] Lagel M.C., Pizzi A., Giovando S., Celzard A. (2014). Development and characterisation of phenolic foams with phenol-formaldehyde-chestnut tannins resin. J. Renew. Mater..

[20] Li C., Wang W., Mu Y., Zhang J., Zhang S., Li J., Zhang W. (2018). Structural properties and copolycondensation mechanism of valonea tannin-modified phenol-formaldehyde resin. J. Polym. Environ..

[21] Li Z., Sutandar E., Goihl T.V., Zhang X., Pan X. (2020). Cleavage of ethers and demethylation of lignin in acidic concentrated lithium bromide (ACLB) solution. Green Chem..

[22] Zhen X., Li H., Xu Z., Wang Q., Xu J., Zhu S., Wang Z., Yuan Z. (2021). Demethylation, phenolation, and depolymerization of lignin for the synthesis of lignin-based epoxy resin via a one-pot strategy. Ind. Crop. Prod..

[23] (2017). Testing Methods for Wood Adhesives and Resins.

[24] (2008). Plastics—Determination of Compressive Properties.

[25] (2022). Plastics—Determination of Thermal Conductivity and Thermal Diffusivity—Part 2: Transient Plane Heat Source (Hot Disc) Method.

[26] (2025). Plastics—Determination of Burning Behaviour by Oxygen Index—Part 4: High Gas Velocity Test.

[27] Alonso M., Oliet M., García J., Rodríguez F., Echeverría J. (2006). Gelation and isoconversional kinetic analysis of lignin-phenol-formaldehyde resol resins cure. Chem. Eng. J..

[28] Pilato L. (2010). Phenolic Resins: A Century of Progress.

[29] Cheng S., D’Cruz I., Yuan Z., Wang M., Anderson M., Leitch M., Xu C.B. (2011). Use of biocrude derived from woody biomass to substitute phenol at a high-substitution level for the production of biobased phenolic resol resins. J. Appl. Polym. Sci..

[30] Xu L.-Y., Shi Z.-G., Feng Y.-Q. (2008). Preparation of a carbon monolith with bimodal perfusion pores. Micropor. Mesopor. Mat..

[31] Hu L., Zhou Y., Zhang M., Liu R. (2011). Characterization and properties of a lignosulfonate-based phenolic foam. Bioresources.

[32] Gao C., Li M., Zhu C., Hu Y., Shen T., Li M., Ji X., Lyu G., Zhuang W. (2021). One-pot depolymerization, demethylation and phenolation of lignin catalyzed by HBr under microwave irradiation for phenolic foam preparation. Compos. Part. B Eng..

[33] Chen G., Liu J., Zhang W., Han Y., Zhang D., Li J., Zhang S. (2021). Lignin-based phenolic foam reinforced by poplar fiber and isocyanate-terminated polyurethane prepolymer. Polymers.

[34] Wang G., Liu X., Zhang J., Sui W., Jang J., Si C. (2018). One-pot lignin depolymerization and activation by solid acid catalytic phenolation for lightweight phenolic foam preparation. Ind. Crop. Prod..

[35] Wang G., Qi S., Xia Y., Parvez A.M., Si C., Ni Y. (2020). Mild one-pot lignocellulose fractionation based on acid-catalyzed biphasic water/phenol system to enhance components’ processability. ACS Sustain. Chem. Eng..

[36] Weng S., Li Z., Bo C., Song F., Xu Y., Hu L., Zhou Y., Jia P. (2023). Design lignin doped with nitrogen and phosphorus for flame retardant phenolic foam materials. React. Funct. Polym..

[37] Melro E., Duarte H., Antunes F.E., Valente A.J., Romano A., Norgren M., Medronho B. (2023). Engineering novel phenolic foams with lignin extracted from pine wood residues via a new levulinic-acid assisted process. Int. J. Biol. Macromol..

[38] Li M., Shan J., Hu Y., Gao C., Long H., Shen T., Tan Z., Zhuang W., Liu D., Zhu C. (2022). Lignin demethylation for modifying halloysite nanotubes towards robust phenolic foams with excellent thermal insulation and flame retardancy. J. Appl. Polym. Sci..

[39] Issaoui H., de Hoyos-Martinez P.L., Pellerin V., Dourges M.-A., Deleuze H., Bourbigo S., Bouhtoury F.C.-E. (2021). Effect of catalysts and curing temperature on the properties of biosourced phenolic foams. ACS Sustain. Chem. Eng..

